# A sampling framework for incorporating quantitative mass spectrometry data in protein interaction analysis

**DOI:** 10.1186/1471-2105-14-299

**Published:** 2013-10-04

**Authors:** George Tucker, Po-Ru Loh, Bonnie Berger

**Affiliations:** 1 Mathematics Department and Computer Science and Artificial Intelligence Laboratory, Massachusetts Institute of Technology, Cambridge, MA, 02139, USA

## Abstract

**Background:**

Comprehensive protein-protein interaction (PPI) maps are a powerful resource for uncovering the molecular basis of genetic interactions and providing mechanistic insights. Over the past decade, high-throughput experimental techniques have been developed to generate PPI maps at proteome scale, first using yeast two-hybrid approaches and more recently via affinity purification combined with mass spectrometry (AP-MS). Unfortunately, data from both protocols are prone to both high false positive and false negative rates. To address these issues, many methods have been developed to post-process raw PPI data. However, with few exceptions, these methods only analyze binary experimental data (in which each potential interaction tested is deemed either observed or unobserved), neglecting quantitative information available from AP-MS such as spectral counts.

**Results:**

We propose a novel method for incorporating quantitative information from AP-MS data into existing PPI inference methods that analyze binary interaction data. Our approach introduces a probabilistic framework that models the statistical noise inherent in observations of co-purifications. Using a sampling-based approach, we model the uncertainty of interactions with low spectral counts by generating an ensemble of possible alternative experimental outcomes. We then apply the existing method of choice to each alternative outcome and aggregate results over the ensemble. We validate our approach on three recent AP-MS data sets and demonstrate performance comparable to or better than state-of-the-art methods. Additionally, we provide an in-depth discussion comparing the theoretical bases of existing approaches and identify common aspects that may be key to their performance.

**Conclusions:**

Our sampling framework extends the existing body of work on PPI analysis using binary interaction data to apply to the richer quantitative data now commonly available through AP-MS assays. This framework is quite general, and many enhancements are likely possible. Fruitful future directions may include investigating more sophisticated schemes for converting spectral counts to probabilities and applying the framework to direct protein complex prediction methods.

## Background

**Figure 1:**
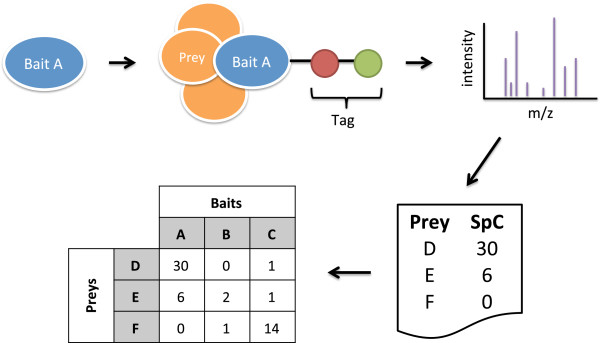
**A typical AP-MS workflow.** A typical AP-MS study consists of performing a set of experiments on *bait proteins* of interest, with the goal of identifying their interaction partners. In each experiment, a bait protein is tagged (e.g., using a FLAG-tag or TAP-tag) and expressed in cells. The bait protein and interacting *prey proteins* are affinity purified. The resulting mixture of bait and bound prey proteins is trypsinized into peptide fragments, which are separated by liquid chromatography and passed to a mass spectrometer for analysis. The mass spectrometer produces intensity spectra, which are matched to peptides to deduce proteins present in the purification. Interacting preys thus identified are assigned semi-quantitative *spectral counts* (SpC) indicating the propensity of each prey to bind to the bait. Data is collated from across the experiments into a matrix of bait-prey spectral counts, which serves as the input to post-processing methods that filter contaminants and identify true interactions.

The importance of protein interactions and protein complexes in understanding cellular functions has driven the generation of comprehensive protein-protein interaction (PPI) maps. The first large-scale PPI maps were generated for the model organism *Saccharomyces cerevisiae*, initially using yeast two-hybrid screens (Y2H) [1,2] and subsequently by affinity purification combined with mass spectrometry (AP-MS, Figure 1) [3,4]. Similarly, high throughput approaches have been applied to comprehensively map the *Drosophila melanogaster* interactome, initially using Y2H [5] and more recently by AP-MS [6]. With advances in experimental protocols and decreasing costs, medium-scale AP-MS studies have become ubiquitous in proteomics for targeted investigation of specific pathways or interactions. The PPI networks these analyses generate have provided exciting insights into biological pathways and protein complexes, e.g., with relevance to human disease [7]. However, raw AP-MS data includes many false positive and false negative interactions, which are serious confounding factors in their interpretation [8,9].

**Figure 2:**
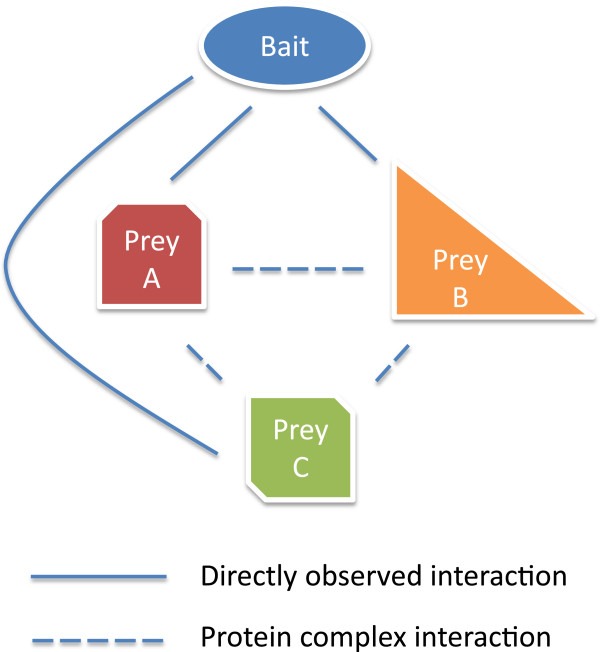
**Direct and indirect interactions in AP-MS data sets.** The diagram depicts a bait protein bound to a prey protein complex. Solid lines indicate bait-prey interactions that could be observed in an AP-MS experiment, while dashed lines indicate prey-prey protein complex interactions that are not directly observable. Spoke methods make predictions only on directly observed interactions (e.g., Bait with Prey A), whereas matrix models infer protein complex interactions (e.g., Prey A with Prey B). Because the prey proteins do not necessarily form a single complex that interacts with the bait, inferences of prey-prey interactions need to be based on the co-occurrence of pairs of preys across many purification experiments, which strengthens the evidence for interaction.

To address these issues, numerous methods have been developed to post-process AP-MS data sets. These generally fall in two classes: spoke and matrix models (Figure 2). Spoke models [10-15] produce confidence scores on bait-prey interactor pairs directly observed in the data (i.e., those with non-zero spectral counts), whereas matrix models [6,9,16-18] additionally infer prey-prey interactions that are not directly observed and hence have broader coverage at the expense of increased false positives. Development of spoke models has been an intense area of research from the outset; see Nesvizhskii [19] for a thorough review. Matrix models rely on analyzing co-occurrences of pairs of proteins across many experiments and were thus less effective on the initial medium-scale AP-MS studies first performed. As larger AP-MS experiments have become more common, however, matrix models have become increasingly relevant because they can leverage the rich co-occurrence information in these data sets. For example, Guruharsha *et al.*[6] reported significantly improved inference on the *Drosophila melanogaster* interactome using a matrix model approach as compared to state-of-the-art spoke methods.

The existing literature on matrix approaches has almost exclusively considered only binary experimental data (i.e., data sets in which bait-prey interactions are deemed either observed or unobserved, with no additional information about propensity of proteins to interact). An exception is the HGSCore method [6], which to our knowledge is the first to use quantitative information from AP-MS experiments in the form of bait-prey spectral counts. In contrast, spoke models have successfully used quantitative information (e.g., spectral counts [10-14,20] and MS1 intensity data [15]) to filter contaminants and assign confidence scores to interactions.

In this study, we propose a novel approach for incorporating quantitative interaction information into AP-MS PPI inference. Our approach aggregates scores over an ensemble of binary data sets that represents the quantitative data, capturing the uncertainty of interactions with low spectral counts. Importantly, the sampling-based framework we propose allows us to directly harness previous binary methods without modification, thus extending previous methods to use quantitative information. We validate our results on a large-scale PPI network and two medium-scale networks. Our approach improves all binary methods that we tested across a broad range of parameter values. In many cases, the improved performance is comparable to or better than state-of-the-art methods that have been developed to leverage spectral counts. Additionally, in the Discussion we characterize previous approaches and identify a common mathematical framework that several successful approaches have used, providing insights that may be valuable in continuing to refine PPI inference techniques.

## Results

### Sampling framework

**Figure 3:**
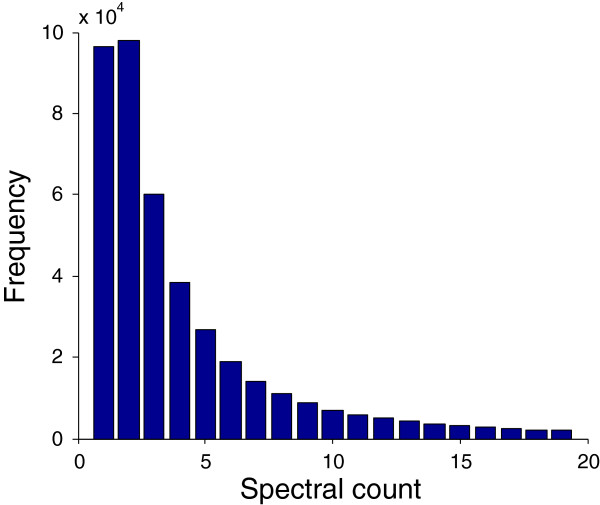
**Histogram of spectral counts in the DPiM data set [**[6]**].** Of 438,557 positive spectral counts, 94% are less than 20 (shown) and nearly half are either 1 or 2. In contrast, the largest spectral count value is 753.

The motivation behind our approach is that spectral count values in AP-MS data sets span a very large dynamic range (from single-digit values to numbers in the thousands - Figure 3), and collapsing this range into binary values—as is necessary to apply several previous methods [9,16-18,21]—loses a great deal of potentially useful information. In particular, our intuition is that bait-prey interactions observed with high spectral counts are much more likely to be true interactions than those with spectral counts of only 1 or 2, which might arise through experimental noise. However, there are exceptions; lower abundance proteins can be true interactors if they are pulled-down reproducibly, and high abundance proteins can be sticky proteins that are not necessarily true interactors.

**Figure 4:**
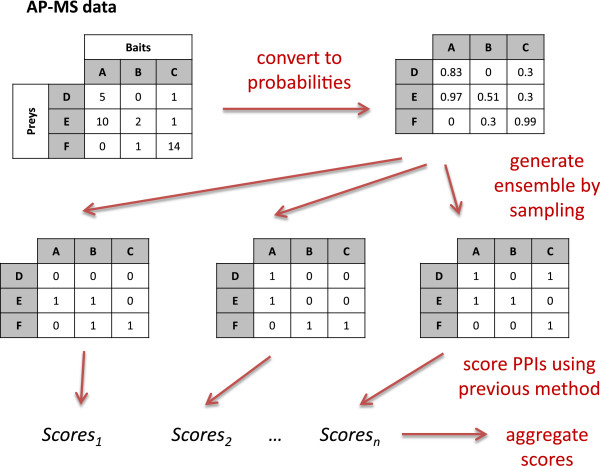
**Sampling approach: Representing spectral counts with ensembles of binary matrices.** A summary of our sampling approach. First, each spectral count in the AP-MS data matrix is converted to a probability 1 - (1 - *p*)^*n*^, where *n* is the spectral count. Then, for each cell of the matrix, we sample an independent Bernoulli random variable according to its probability. We repeat this procedure independently for a desired number of trials, obtaining an ensemble of binary matrices representing the original quantitative AP-MS data. Each binary matrix is then used as input to a PPI inference method of choice that operates on binary data, and the results from each trial are aggregated to produce an ensemble score. Notably, the existing PPI inference method is directly applied to each binary matrix without modification.

To model this uncertainty in the bait-prey interaction data in a way that allows us to harness existing methods that operate on binary data, we propose a sampling framework that represents the quantitative (spectral count) data set using an ensemble of binary data sets (Figure 4). We do so by first converting each positive spectral count into a probability that represents the confidence that the observed interactions were not experimental artifacts. Then, for each of a specified number of trials, we create a binary data set by sampling bait-prey interactions according to their probabilities, and we apply the existing method to the binary data set. Finally, we aggregate the results over the ensemble to produce an overall ranking of possible PPIs.

Explicitly, our framework takes as input a matrix of spectral counts (*n*_*ij*_), where columns correspond to purification experiments and rows to prey proteins. We convert a spectral count of *n* to the probability 1 - (1 - *p*)^*n*^, where *p* is a user-defined parameter representing the probability that a single spectral count is the result of a true observation, and we view the *n* observed spectral counts as arising independently. Using these probabilities, we generate binary data sets of the same size as the original spectral count input matrix by putting a 1 in each matrix cell independently with probability 1 - (1 - *p*)^*n*^_*ij*_. The resulting distribution of alternative binary realizations of the spectral count matrix thus reflects the range of confidences in different bait-prey interactions, in contrast to the common approach of converting the spectral count matrix to a single binary matrix simply by replacing all positive spectral counts with 1s.

Given an ensemble of alternative binary realizations and an existing PPI scoring algorithm that operates on binary data, we apply the PPI scoring algorithm to each realization, in each case producing a score for every possible PPI. We then produce an aggregate score for every PPI by taking the mean of the ensemble of scores for that PPI, possibly after applying an appropriate transformation. (A slight subtlety can arise in aggregating scores because depending on the shape of the score distribution, taking the mean may not be robust. Among the algorithms we evaluated, we observed that the SAI score [21] could produce unbounded negative values, so we lower-bounded SAI scores at 0 before aggregation in order to prevent a single realization from having an extreme effect on the ensemble score.)

An additional consideration is the size of the ensemble required to produce stable results. In the tests we describe below, we ran 120 independent trials and found reasonable score separation between low, medium and high confidence interactions (Additional file 1: Figure S7). Then we further verified that increasing the ensemble size by a factor of four had a negligible impact on the results, indicating that 120 trials was sufficient to average out the stochasticity of the method. Although the minimum number of trials required will vary with the specific data set, our experiments suggest that in general, such a number of trials should sufficiently explore the space of binary realizations without presenting a computational burden, especially because the ensemble computations can be easily parallelized.

### Validation on three AP-MS data sets

We benchmarked our method by producing predictions from three AP-MS data sets: the recently published Drosophila Protein interaction Map (DPiM) [6] which includes over 3000 baits, a medium-scale human data set (TIP49) with 27 baits [10], and a *Drosophila* study focusing on the MAPK pathway with 21 baits [14]. On each evaluation data set, we applied our sampling framework to three previously published binary matrix methods for PPI inference: Hart et al. [16], PE [9], and SAI [21]. Each method produced a ranked list of interactions.

A standard approach to evaluating inferred interactions is to compare predictions with a high-confidence gold standard set. However, such a reference is challenging to construct. Few large-scale databases are available, and even the largest are understood to be incomplete and include false positive interactions. In light of these concerns, we follow the validation strategy used in Guruharsha et al. [6] of considering the overlaps between multiple curated data sets, obtaining subsets of PPIs with increasingly stringent thresholds on the number of supporting sources. The idea is that we can have high confidence in interactions supported by multiple lines of medium-confidence evidence, reducing the false positive rate in the gold standard data set (with the caveat that this approach may be biased toward well-studied pathways). We applied this procedure to create validation data sets from the Drosophila Interactions Database (DroID) [22] for *Drosophila* PPI predictions and BioGRID [23] for human PPI predictions. (See Methodsfor details.)

**Figure 5:**
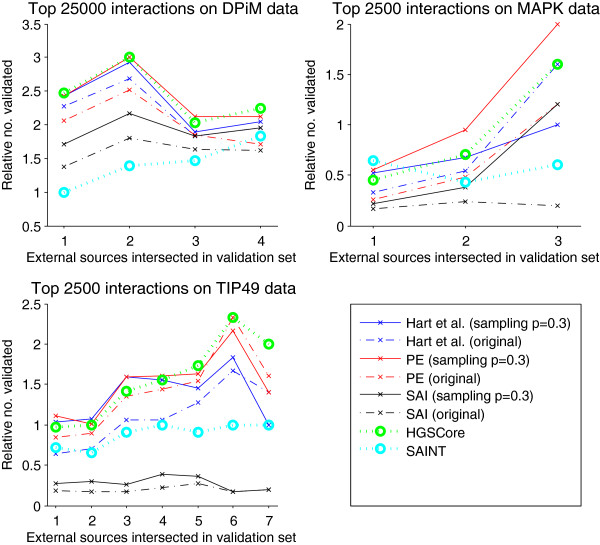
**Performance comparison of PPI inference methods.** Performance of our sampling approach applied to PPI inference methods that operate on binary bait-prey interaction data (Hart et al. [16], PE [9], and SAI [21]), and compared to state-of-the-art methods that make use of spectral counts (HGSCore [6] and SAINT [13]). For each method that operates on binary data, two curves are plotted: (i) a dashed curve that shows the performance of the method when applied to a direct binarization of the spectral count data (i.e., converting all nonzero spectral counts to 1s)—a common approach—and (ii) a solid curve showing performance upon applying our sampling approach with *p* = 0.3. We evaluate performance according to the number of PPI inferences (out of the highest-confidence 25,000 or 2,500) validated on gold standard tests, as explained in the main text. The plot shows performance relative to a baseline method of simply ranking PPIs in decreasing order of observed spectral counts. All methods were run using default parameter settings.

For each method, we compared the top 25,000 predicted interactions for the DPiM data set and the top 2,500 predicted interactions for the TIP49 and MAPK data sets to gold standard interactions supported by increasing numbers of sources, as in Guruharsha et al. [6]. Our sampling framework produced robust improvement to the binary methods across all levels of support and all data sets (Figure 5). Moreover, the improved methods perform better than or comparably to state-of-the-art methods that use spectral count data (HGSCore [6] and SAINT [13]). The choice of cutoff at the top 25,000 and 2,500 interactions was arbitrary, and the results are similar at different cutoffs (Additional file 1: Figures S1, S2, S3).

**Figure 6:**
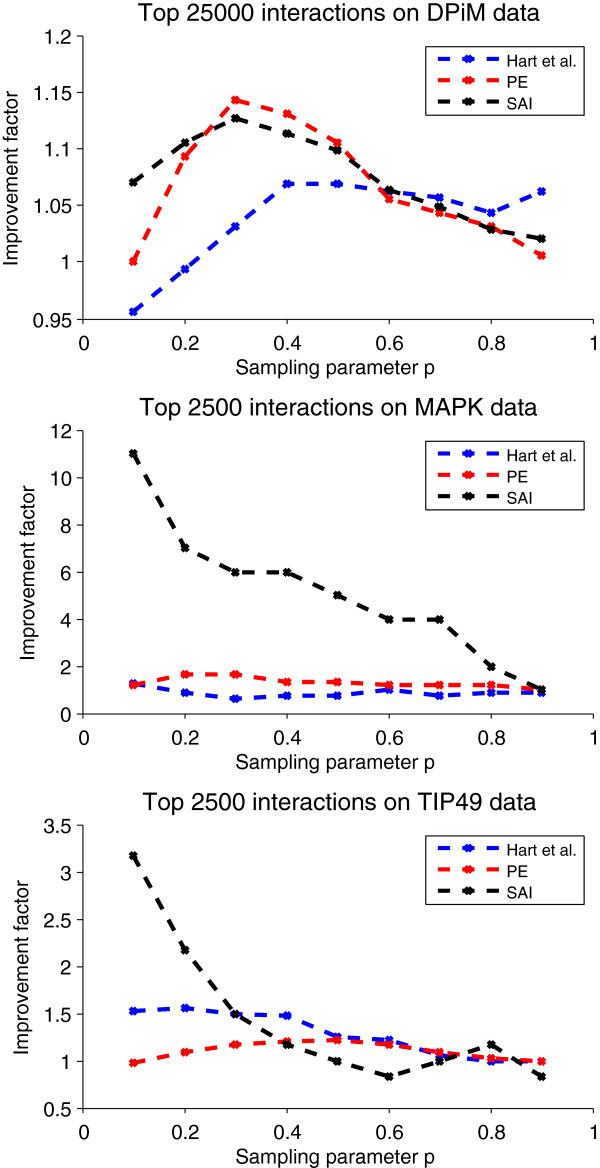
**Sensitivity of performance to sampling parameter*p*.** We plot the improvement in performance, as a function of *p*, achieved by applying our sampling approach vs. applying methods to direct binarizations of spectral count data. Performance is measured using the same setup as in Figure 5. For figure readability, we show results for just the validation sets consisting of interactions supported by at least 3 pieces of evidence; similar results hold for the other validation sets.

The sole parameter in our method is the probability *p* that represents the reliability of a single peptide observation. We suggest a default value of *p* = 0.3, but the performance improvements obtained using our sampling framework are robust across a wide range of values of *p* (Figure 6; Additional file 1: Figures S4, S5) and for different confidence cutoffs (Additional file 1: Figure S6).

## Discussion

The literature of published methods for PPI inference from AP-MS data is substantial, and in continuing to develop methodological improvements, it is valuable to understand the similarities and differences among existing approaches and identify key ideas.

### Characterization of methods

Broadly speaking, methods can be broken down into two classes of models—spoke and matrix models—and by their scoring method. Spoke models make predictions solely on bait-prey interactions, while matrix models infer prey-prey interactions as well. Because prey-prey relationships are never directly observed, matrix models use the co-occurrence of pairs of proteins over multiple experiments to make inferences. Methods can also be characterized by their scoring functions, which generally fall into two classes: evidence-based scoring and null model-based scoring. In evidence-based scoring, models are built that estimate the likelihoods of observations under interacting and non-interacting pairs. Typically, a log likelihood ratio is then summed across experiments, implicitly assuming independence. Evidence-based scoring approaches, such as the PE [9] and C2S [18] scores, can easily combine direct bait-prey observations and prey-prey observations in the same model. However, because likelihood models for interacting and for non-interacting pairs must be constructed, these scores tend to have more tuning parameters that must be estimated from scarce gold standard validation data. In null model based approaches, such as Hart et al. [16], HGSCore [6], and SAI [21], a model for non-interacting pairs is assumed and fit from the data. This forms an empirical null distribution under which observations can be scored. The advantage of such an approach is that only the null distribution has to be tuned, so in many cases tuning with gold standard validation sets is unnecessary.

An additional consideration for any method that combines spoke and matrix information is the balance between information from direct bait-prey observations and prey-prey co-occurrences. These sources of information are clearly distinct, so the weighting between the two must be carefully calibrated, potentially requiring gold standard validation data. Proper calibration is critical to performance and may explain why Hart et al. and HGSCore, which seemingly sub-optimally ignore spoke information, perform significantly better on our tests than SAI [21], which uses both spoke and matrix information.

For experiments with a handful of baits, we expect that methods relying on spoke information will have the best performance because matrix methods rely on analyzing co-occurrences of pairs of proteins across many experiments. However, even for the medium-scale experiments that we analyzed, methods that rely solely on matrix information performed competitively with methods that used spoke information. We foresee that as experiment sizes grow, matrix relationships will be increasingly informative, so it will be crucial to consider both spoke and matrix information. Although our approach is applicable to any binary method, in our experiments, we found that for nearly all experiments PE was the top performer amongst the binary methods. In addition, because PE uses spoke and matrix information, we recommend using it in our framework.

### Low rank plus sparse matrix framework

Interestingly, several methods (e.g., Hart et al. [16], HGSCore [6], SAINT [13]) can be understood under a common “low rank plus sparse matrix” framework. Hart et al. [16] considered a null model in which interaction partners are chosen independently at random in proportion to the number of interactions each partner protein was observed in. Although Hart et al. [16] used a hypergeometric distribution, for large-scale studies, the score for interaction between proteins *A* and *B* is well approximated using a Poisson cumulative distribution function (CDF), taking the form

$$\;-\log\left(1-\text{PoissonCDF}\left(X_{\text{AB}};\lambda =\frac{N_{A}}{N}\times \frac{N_{B}}{N}\times N\right)\right),$$

where *X*_*AB*_ is the number of experiments that protein *A* and protein *B* co-purify in, *N*_*A*_ (resp. *N*_*B*_) is the number of co-purifying pairs that protein *A* (resp. *B*) is observed in, and *N* is the total number of co-purifying pairs.

In the above form, *λ* factors as a rank-1 matrix, so that the method can be seen as modeling the co-occurrence matrix *X*_*AB*_as the sum of a rank-1 “background” matrix (blurred by Poisson noise) and a sparse matrix indicating true interactions. Notably, *X*_*AB*_ignores quantitative information, simply counting experiments in which proteins were co-purified. HGSCore [6] is an extension of the Hart et al. score that incorporates spectral count information through a transformation of the spectral counts (instead of directly using the co-occurrence matrix) and then analyzes the pseudo co-occurrence matrix in a similar manner. For the same reasons as above, we can view HGSCore as a rank-1 null model plus sparse true interactions, where the rank-1 component is estimated from a transformation of the spectral count data.

Similarly, SAINT [13] uses a probabilistic formulation to decompose a matrix of observed counts as a sum of: a rank-1 matrix, a sparse true interaction matrix, and generalized Poisson noise. Interestingly, SAINT decomposes the matrix of spectral counts—as opposed to co-occurrences—and has an entirely different justification for using a low rank model. Hart et al. and HGSCore assume that interaction partners are chosen at random in the null model, which gives rise to a low rank structure in the co-occurrence observations. Alternatively, SAINT assumes that contaminant proteins produce similar spectral counts across all bait experiments, which gives rise to a low rank structure in the spectral count observations. SAINT uses solely spoke evidence while Hart et al. and HGSCore use only co-occurrence evidence, suggesting that some combination of these approaches under a common framework may be an interesting direction for future investigation.

### Moving toward complexes

As protein biology is ultimately driven by the interactions of protein complexes—not just pairwise protein interactions—recent work has begun inferring protein complexes directly from AP-MS data [10,24-28]. Traditionally, methods have first inferred PPIs and then clustered proteins into complexes (e.g., Guruharsha et al. [6]); however, information may be lost in this two-step procedure that first post-processes the data into high-confidence pairwise interactions. As with matrix models, some recent methods that bypass this first step have considered only binary experimental data [24,25], whereas others have successfully used spectral count information [10,26-28]. A similar sampling approach could be used to extend methods that consider only binary data to leverage spectral counts.

## Conclusions

As large-scale AP-MS experiments have become more common, an opportunity to leverage indirect co-occurrence information for PPI inference has arisen. Our sampling framework harnesses existing matrix methods for PPI inference that could previously only be applied to binary interaction data, achieving robust improvements across a range of data sets and enabling comparable or better performance versus current state-of-the-art methods. This framework extends the existing body of work on binary interaction analysis to apply to richer spectral count data now commonly available. Moreover, it is sufficiently general to have potential for future application in related protein interaction inference studies.

## Methods

### AP-MS data sets

The main data set we analyzed, DPiM, is a large-scale AP-MS study of the *Drosophila* proteome with 3485 experiments, which collectively pulled down 4927 distinct proteins ([6], Table S1). The DPiM data set is unique among publicly available AP-MS data sets because of its large size, which gives us confidence that the results we observed are not the result of random noise or overfitting. We also tested our approach on two medium-scale AP-MS data sets. One is another *Drosophila* study that focused on the MAPK pathway [14]; this data set contained 63 experiments, which collectively pulled down 1078 distinct proteins and included 9 control experiments. The other is a human data set referred to as TIP49 and originally published in Sardiu et al. [10]. We obtained the interaction data set, consisting of 35 experiments, which collectively pulled down 1207 distinct proteins and included 9 control experiments, from Choi et al. ([13], Table S1).

### Validation data sets

To validate *Drosophila* PPI inferences, we used the data sets in the DroID database [22]. We excluded the Perrimon co-AP complex and DPiM co-AP complex data sets to avoid contaminating our test sets with training data, leaving 7 other PPI data sets that we used in the above validation procedure. The validation set contained 58,657 interactions supported by at least one source, 3,310 interactions supported by at least two sources, 289 interactions supported by at least three sources, and 67 interactions supported by at least four sources.

To validate human PPI inferences, we used BioGRID v3.1.79 [23], which contains 40,680 interactions supported by at least one source, 11,054 interactions supported by at least two sources, 4,879 interactions supported by at least three sources, and 2,271 interactions supported by at least four sources.

### Implementation

We re-implemented the SAI [21], PE [9], Hart et al. [16], and HGSCore [6] methods; each is described in its reference but code is not provided. The PE score uses two parameters, *r*, representing the probability of detecting a true association in a purification experiment, and *n*_pseudo_, the number of pseudocounts added for each prey. Since Collins et al. [9] estimates values of *r* = 0.51,0.62, and 0.265 on three example data sets and suggests using *n*_pseudo_ = 20,10, or 5, we set *r* = 0.3 and *n*_pseudo_ = 10. We downloaded and ran SAINT [13] with default parameters.

We also implemented the C2S score [18] but found its performance to be highly sensitive to the *tpr* (true positive rate) parameter; some values of *tpr*—including the default 0.6 in at least one of our tests—result in inferred values of the probabilistic parameters *r*_*bp*_and *r*_*pp*_ that exceed 1, causing improper values in subsequent calculations (e.g., logarithms of negative numbers). We therefore excluded C2S from our analysis.

When we applied our sampling framework to data sets containing replicates, we treated columns corresponding to replicates independently. When we tested all of the methods on data sets containing controls, only SAINT, which explicitly models control data, used the controls.

## Availability of supporting data

The C++ code for our implementations is provided in Additional file 2.

## Competing interests

The authors declare that they have no competing interests.

## Authors’ contributions

GT and PL implemented and tested the methods; BB guided this research. All authors participated in the design of the project and writing of the manuscript. All authors read and approved the final manuscript.

## Supplementary Material

Additional file 1 Supplementary figures. Click here for file

Additional file 2 **C++ implementation.** We have included the C++ code for our implementations and documentation in a zipped archive file. The archive contains a readme file with instructions to compile the code and several sample files to illustrate usage. Click here for file

## References

[B1] Uetz P Giot L Cagney G Mansfield TA Judson RS Knight JR Lockshon D Narayan V Srinivasan M Pochart P Qureshi-Emili A Li Y Godwin B Conover D Kalbfleisch T Vijayadamodar G Yang M Johnston M Fields S Rothberg JM A comprehensive analysis of protein–protein interactions in Saccharomyces cerevisiae Nature 2000 403 6770 623 627 10.1038/35001009 10688190

[B2] Ito T Chiba T Ozawa R Yoshida M Hattori M Sakaki Y A comprehensive two-hybrid analysis to explore the yeast protein interactome Proc Natl Acad Sci 2001 98 8 4569 4574 10.1073/pnas.061034498 11283351 PMC31875

[B3] Gavin AC Bösche M Krause R Grandi P Marzioch M Bauer A Schultz J Rick JM Michon AM Cruciat CM Remor M Höfert C Schelder M Brajenovic M Ruffner H Merino A Klein K Hudak M Dickson D Rudi T Gnau V Bauch A Bastuck S Huhse B Leutwein C Heurtier MA Copley RR Edelmann A Querfurth E Rybin V Functional organization of the yeast proteome by systematic analysis of protein complexes Nature 2002 415 6868 141 147 10.1038/415141a 11805826

[B4] Ho Y Gruhler A Heilbut A Bader GD Moore L Adams SL Millar A Taylor P Bennett K Boutilier K Yang L Wolting C Donaldson I Schandorff S Shewnarane J Vo M Taggart J Goudreault M Muskat B Alfarano C Dewar D Lin Z Michalickova K Willems AR Sassi H Nielsen PA Rasmussen KJ Andersen JR Johansen LE Hansen LH Systematic identification of protein complexes in Saccharomyces cerevisiae by mass spectrometry Nature 2002 415 6868 180 183 10.1038/415180a 11805837

[B5] Giot L Bader JS Brouwer C Chaudhuri A Kuang B Li Y Hao YL Ooi CE Godwin B Vitols E Vijayadamodar G Pochart P Machineni H Welsh M Kong Y Zerhusen B Malcolm R Varrone Z Collis A Minto M Burgess S McDaniel L Stimpson E Spriggs F Williams J Neurath K Ioime N Agee M Voss E Furtak K A protein interaction map of Drosophila melanogaster Science 2003 302 5651 1727 1736 10.1126/science.1090289 14605208

[B6] Guruharsha KG Rual JF Zhai B Mintseris J Vaidya P Vaidya N Beekman C Wong C Cenaj O McKillip E Shah S Stapleton M Yu C Parsa B Chen X Kapadia B VijayRaghavan K Artavanis-Tsakonas S A protein complex network of Drosophila melanogaster Cell 2011 147 3 690 703 10.1016/j.cell.2011.08.047 22036573 PMC3319048

[B7] Jäger S Cimermancic P Gulbahce N Johnson JR McGovern KE Clarke SC Shales M Mercenne G Pache L Li K Hernandez H Jang GM Roth SL Akiva E Marlett J Stephens M D’Orso I Fernandes J Fahey M Mahon C O’Donoghue AJ Todorovic A Morris JH Maltby DA Alber T Cagney G Bushman FD Young JA Chanda SK Sundquist WI Global landscape of HIV-human protein complexes Nature 2011 481 7381 365 370 22190034 10.1038/nature10719 PMC3310911

[B8] Cargile BJ Bundy JL Stephenson JL Potential for false positive identifications from large databases through tandem mass spectrometry J Proteome Res 2004 3 5 1082 1085 10.1021/pr049946o 15473699

[B9] Collins SR Kemmeren P Zhao XC Greenblatt JF Spencer F Holstege FC Weissman JS Krogan NJ Toward a comprehensive atlas of the physical interactome of Saccharomyces cerevisiae Mol Cell Proteomics 2007 6 3 439 450 17200106 10.1074/mcp.M600381-MCP200

[B10] Sardiu ME Cai Y Jin J Swanson SK Conaway RC Conaway JW Florens L Washburn MP Probabilistic assembly of human protein interaction networks from label-free quantitative proteomics Proc Natl Acad Sci 2008 105 5 1454 1459 10.1073/pnas.0706983105 18218781 PMC2234165

[B11] Sowa ME Bennett EJ Gygi SP Harper JW Defining the human deubiquitinating enzyme interaction landscape Cell 2009 138 2 389 403 10.1016/j.cell.2009.04.042 19615732 PMC2716422

[B12] Lavallée-Adam M Cloutier P Coulombe B Blanchette M Modeling contaminants in AP-MS/MS experiments J Proteome Res 2010 10 2 886 895 21117706 10.1021/pr100795z PMC4494835

[B13] Choi H Larsen B Lin ZY Breitkreutz A Mellacheruvu D Fermin D Qin ZS Tyers M Gingras AC Nesvizhskii AI SAINT: probabilistic scoring of affinity purification-mass spectrometry data Nat Methods 2010 8 70 73 21131968 10.1038/nmeth.1541 PMC3064265

[B14] Sun X Hong P Kulkarni M Kwon Y Perrimon N An advanced method for identifying protein-protein interaction by analyzing TAP/MS data IEEE International Conference on Bioinformatics and Biomedicine (BIBM) 2012 Los Alamitos, CA, USA: IEEE Computer Society 1 6

[B15] Choi H Glatter T Gstaiger M Nesvizhskii AI SAINT-MS1: Protein–Protein Interaction Scoring Using Label-free Intensity Data in Affinity Purification-Mass Spectrometry Experiments J Proteome Res 2012 11 4 2619 2624 10.1021/pr201185r 22352807 PMC3744231

[B16] Hart GT Lee I Marcotte EM A high-accuracy consensus map of yeast protein complexes reveals modular nature of gene essentiality BMC Bioinformatics 2007 8 236 10.1186/1471-2105-8-236 17605818 PMC1940025

[B17] Yu X Ivanic J Wallqvist A Reifman J A novel scoring approach for protein co-purification data reveals high interaction specificity PLoS Comput Biol 2009 5 9 e1000515 10.1371/journal.pcbi.1000515 19779545 PMC2738424

[B18] Xie Z Kwoh CK Li XL Wu M Construction of co-complex score matrix for protein complex prediction from AP-MS data Bioinformatics 2011 27 13 i159—i166 21685066 10.1093/bioinformatics/btr212 PMC3117344

[B19] Nesvizhskii AI Computational and informatics strategies for identification of specific protein interaction partners in affinity purification mass spectrometry experiments Proteomics 2012 12 10 1639 1655 10.1002/pmic.201100537 22611043 PMC3744239

[B20] Friedman AA Tucker G Singh R Yan D Vinayagam A Hu Y Binari R Hong P Sun X Porto M Pacifico S Murali T Jr. Finley R Asara JM Berger B Perrimon N Proteomic and functional genomic landscape of receptor tyrosine kinase and ras to extracellular signal-regulated kinase signaling Sci Signal 2011 4 196 rs10 10.1126/scisignal.2002029 22028469 PMC3439136

[B21] Gavin AC Aloy P Grandi P Krause R Boesche M Marzioch M Rau C Jensen LJ Bastuck S Dümpelfeld B Edelmann A Heurtier MA Hoffman V Hoefert C Klein K Hudak M Michon AM Schelder M Schirle M Remor M Rudi T Hooper S Bauer A Bouwmeester T Casari G Drewes G Neubauer G Rick JM Kuster B Bork P Proteome survey reveals modularity of the yeast cell machinery Nature 2006 440 7084 631 636 10.1038/nature04532 16429126

[B22] Murali T Pacifico S Yu J Guest S Roberts GG Finley RL DroID 2011: a comprehensive, integrated resource for protein, transcription factor, RNA and gene interactions for Drosophila Nucleic Acids Res 2011 39 suppl 1 D736 D743 21036869 10.1093/nar/gkq1092 PMC3013689

[B23] Stark C Breitkreutz BJ Reguly T Boucher L Breitkreutz A Tyers M BioGRID: a general repository for interaction datasets Nucleic Acids Res 2006 34 suppl 1 D535 D539 16381927 10.1093/nar/gkj109 PMC1347471

[B24] Zhang B Park BH Karpinets T Samatova NF From pull-down data to protein interaction networks and complexes with biological relevance Bioinformatics 2008 24 7 979 986 10.1093/bioinformatics/btn036 18304937

[B25] Geva G Sharan R Identification of protein complexes from co-immunoprecipitation data Bioinformatics 2011 27 111 117 10.1093/bioinformatics/btq652 21115439 PMC3008648

[B26] Sardiu ME Florens L Washburn MP Evaluation of clustering algorithms for protein complex and protein interaction network assembly J Proteome Res 2009 8 6 2944 2952 10.1021/pr900073d 19317493

[B27] Choi H Kim S Gingras AC Nesvizhskii AI Analysis of protein complexes through model-based biclustering of label-free quantitative AP-MS data Mol Syst Biol 2010 6 1 11 10.1038/msb.2010.41 PMC2913403 20571534

[B28] Stukalov A Superti-Furga G Colinge J Deconvolution of targeted protein–protein interaction maps J Proteome Res 2012 11 8 4102 4109 10.1021/pr300137n 22724552

