# Supplementary material for: A sampling framework for incorporating quantitative mass spectrometry data in protein interaction analysis
Source: BMC Bioinformatics. 2013 Oct 4;14:299. doi: 10.1186/1471-2105-14-299 (PMC3851523; doi:10.1186/1471-2105-14-299)
Supplement: Additional file 1 — Supplementary figures. [file 1471-2105-14-299-S1.pdf]

Additional Files

Additional file 1 — Supplementary figures

Figure S1: As the validation databases for protein interactions are not complete, we do not have true negative protein interactions, so we cannot form ROC curves. We, as is typical (see [12, 13]), plot percent of predicted interactions present in the respective validation set for a varying number of predicted interactions, which conveys conceptually similar information to a ROC curve. For this figure, all interactions supported by at least one external source are included in the validation set. Otherwise, the setup is the same as Figure 5.

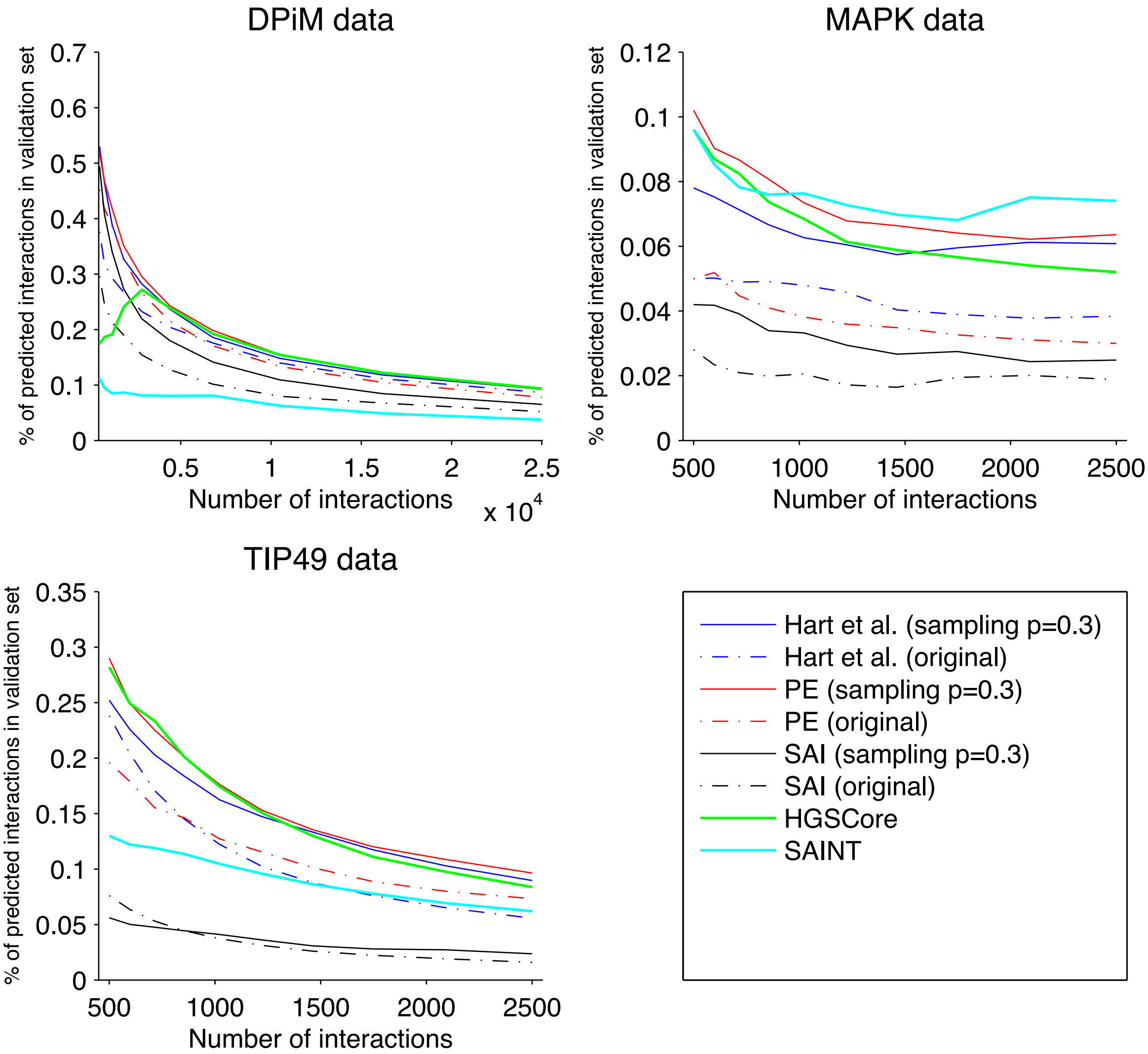

Figure S2: Percent of predicted interactions present in the respective validation set for a varying number of predicted interactions. For this figure, all interactions supported by at least two external sources are included in the validation set. Otherwise, the setup is the same as Figure 5.

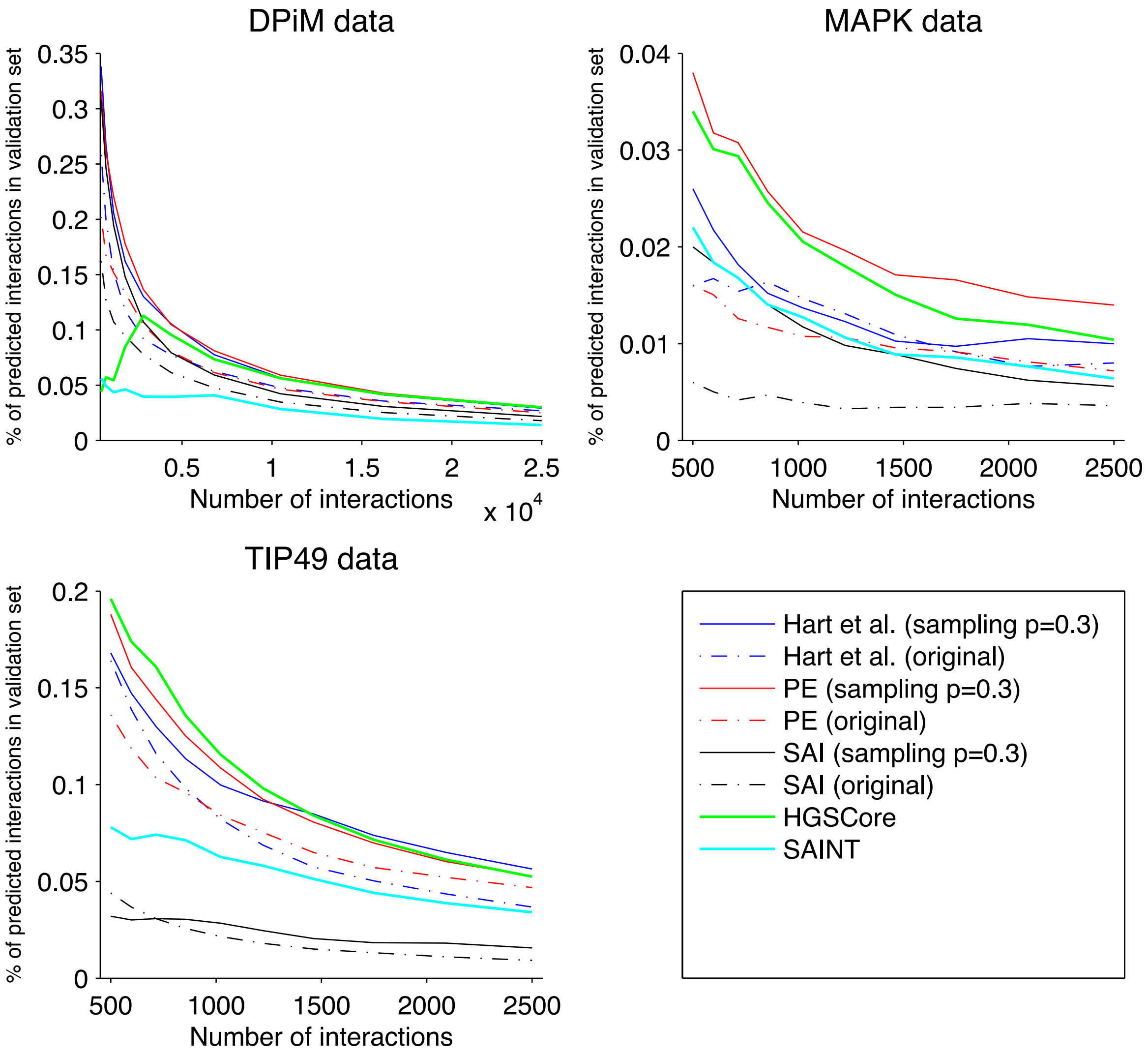

Figure S3: Percent of predicted interactions present in the respective validation set for a varying number of predicted interactions. For this figure, all interactions supported by at least three external sources are included in the validation set. Otherwise, the setup is the same as Figure 5.

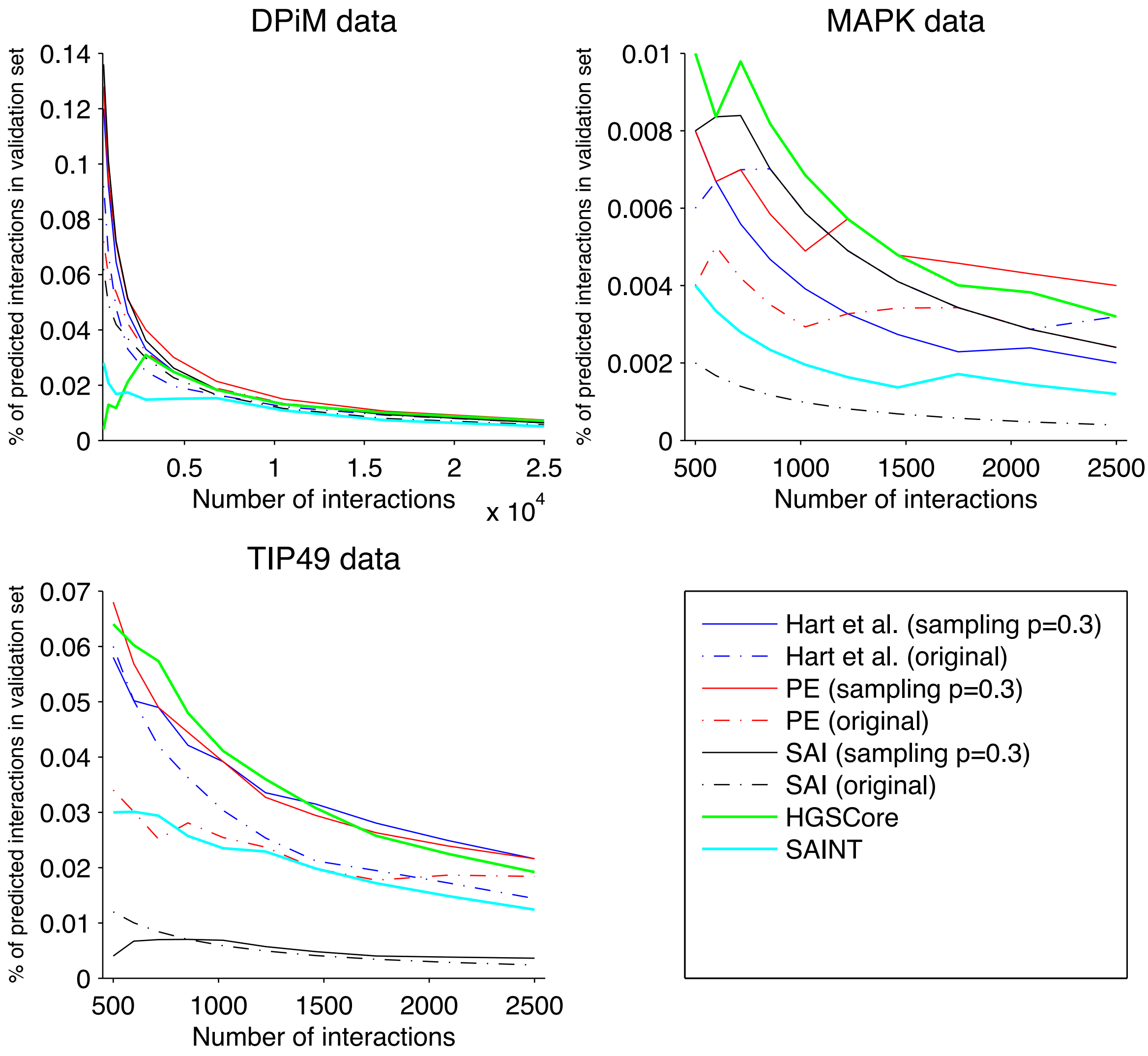

Figure S4: Performance comparison of methods using $p = 0.2$ as the sampling parameter. The setup is otherwise the same as in Figure 5.

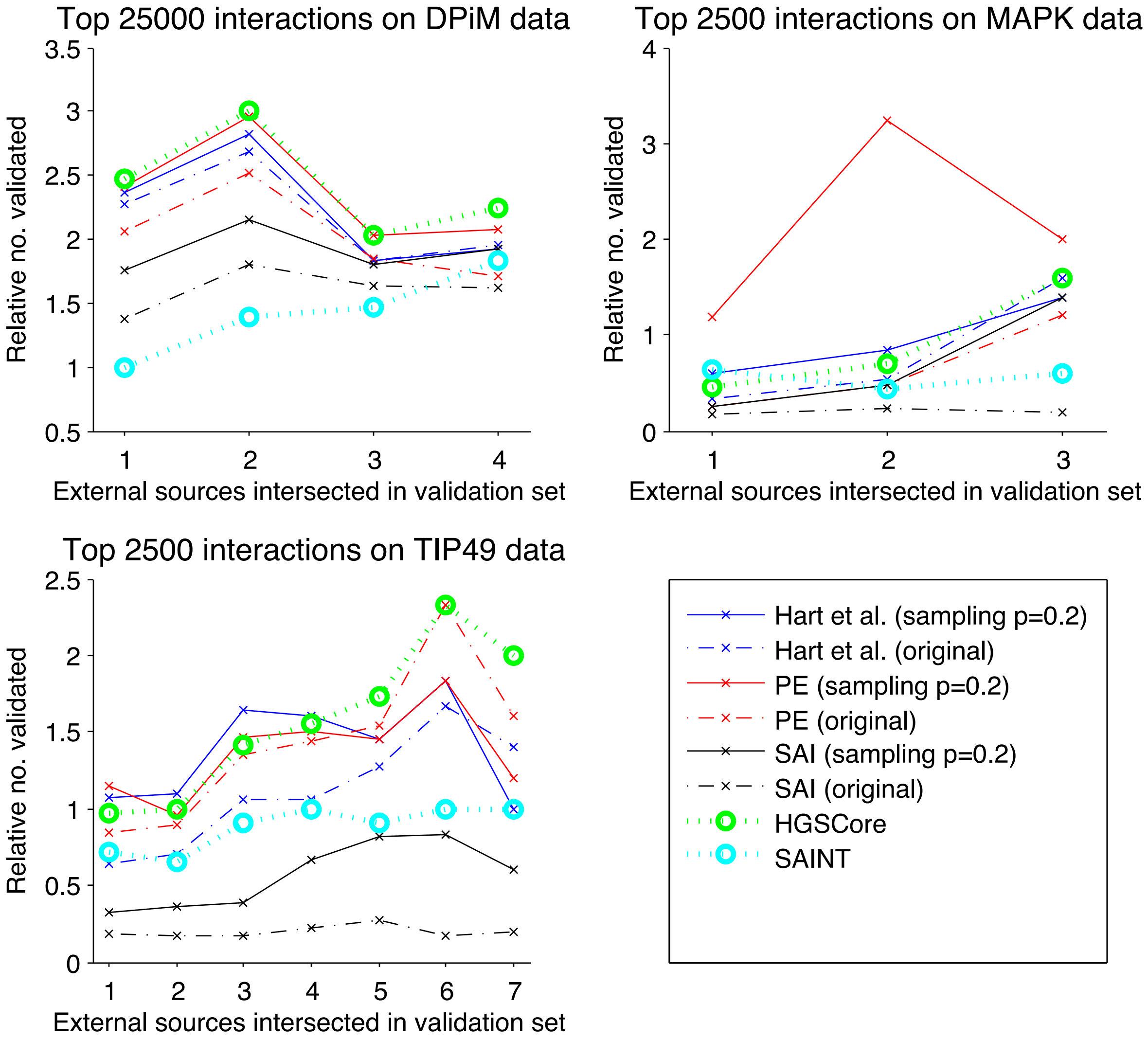

Figure S5: Performance comparison of methods using $p = 0.5$ as the sampling parameter. The setup is otherwise the same as in Figure 5.

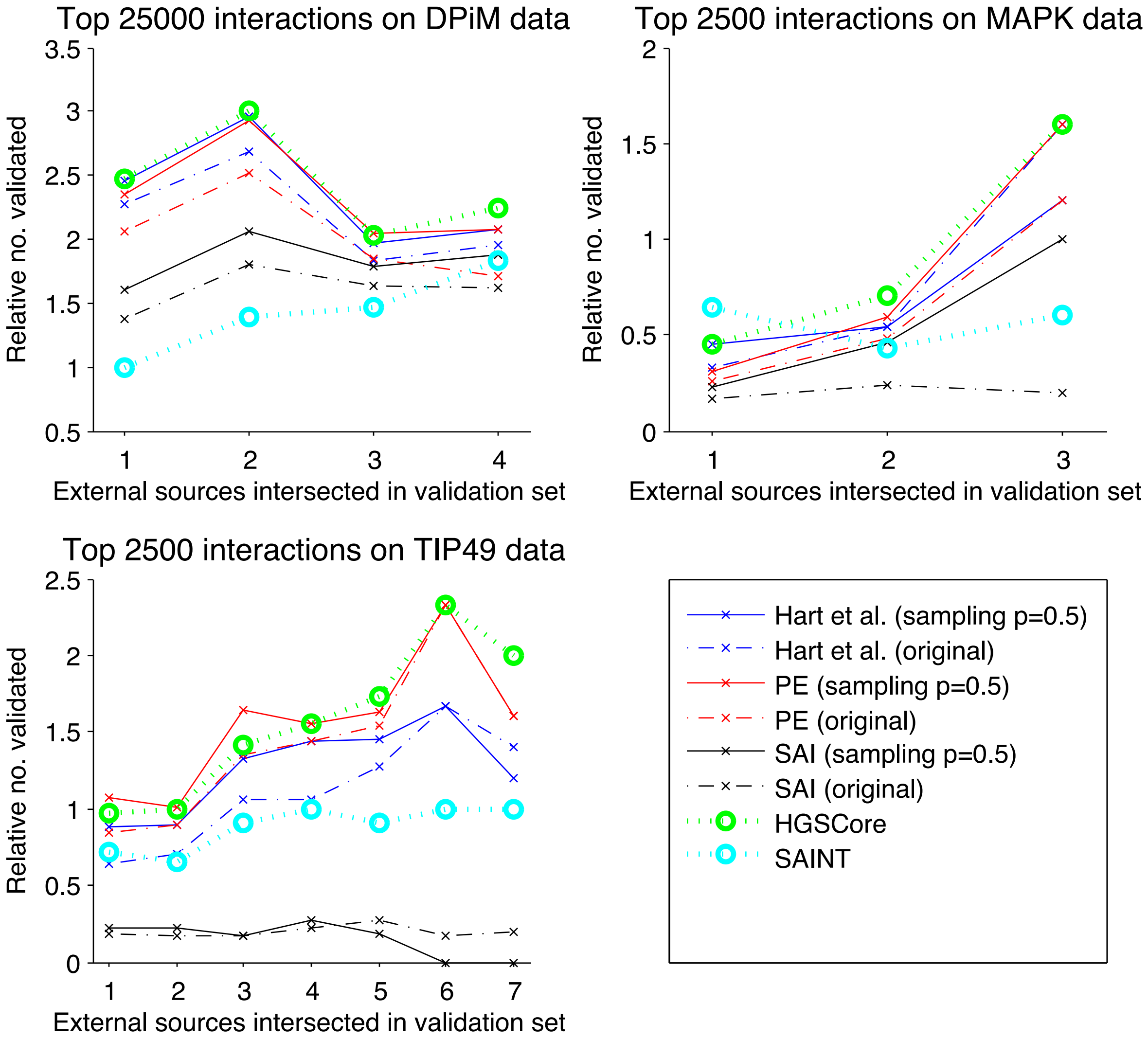

Figure S6: Sensitivity of performance to sampling parameter p for higher-confidence predictions. Only the top 40% of predictions considered in Figure 6 are evaluated here. The setup is otherwise the same.

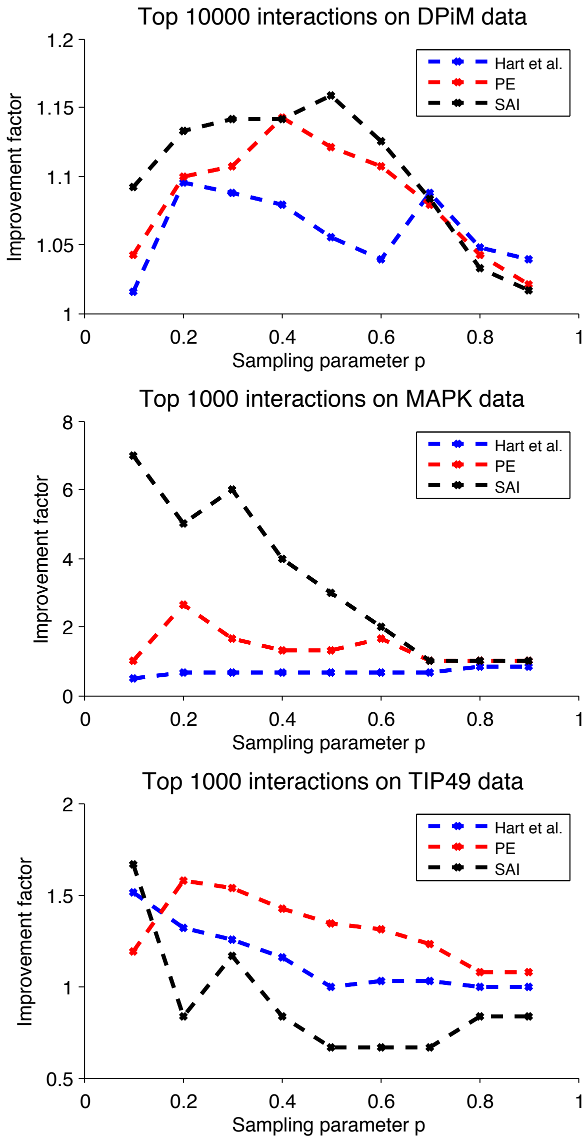

Figure S7: Distribution of scores over multiple binary realizations representative interactions. The score distributions for low, medium and high confidence interactions overlap slightly indicating the importance of averaging over multiple samples.

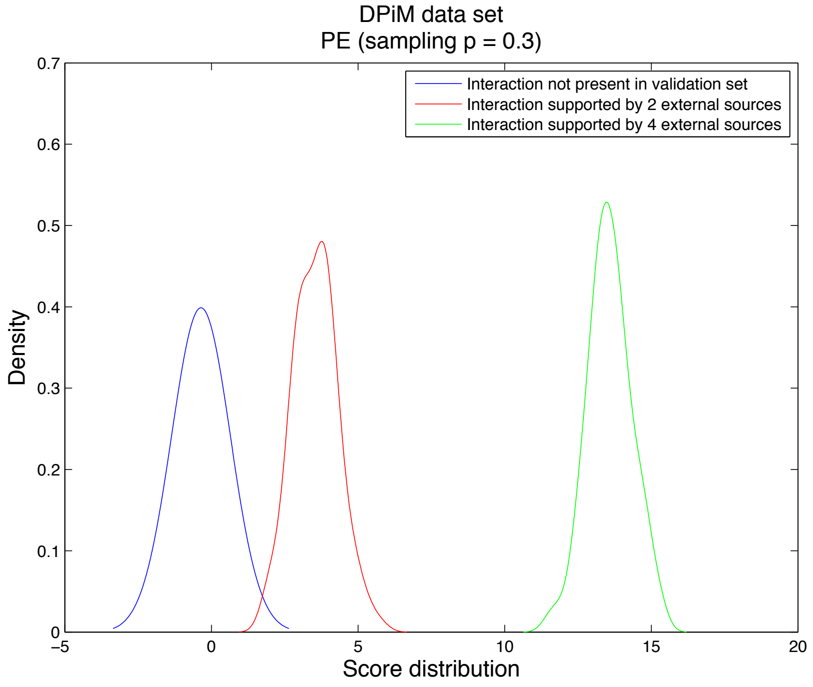

Additional file 2 — C++ implementation

We have included the C++ code for our implementations and documentation in a zipped archive file. The archive contains a readme file with instructions to compile the code and several sample files to illustrate usage.
